# Psychometric properties of instrumented postural sway measures recorded in community settings in independent living older adults

**DOI:** 10.1186/s12877-020-1489-0

**Published:** 2020-02-28

**Authors:** Bader A. Alqahtani, Patrick J. Sparto, Susan L. Whitney, Susan L. Greenspan, Subashan Perera, Jennifer S. Brach

**Affiliations:** 1 Department of Health and Rehabilitation sciences, Prince Sattam Bin Abdulaziz University, Al-Kharj, 11942 Kingdom of Saudi Arabia; 2 Department of Physical Therapy, University of Pittsburgh, Bridgeside Point 1, 100 Technology Drive, Suite 210, Pittsburgh, PA 15219 USA; 3 Department of Medicine, University of Pittsburgh, 3471 Fifth Avenue, Kaufmann Medical Building, Suite 500, Pittsburgh, PA 15213 USA

**Keywords:** Accelerometer, Independent living facilities, Older adults, Standing balance

## Abstract

**Background:**

In the last few decades, research related to balance in older adults has been conducted in lab-based settings. The lack of portability and high cost that is associated with the current gold standard methods to quantify body balance limits their application to community settings such as independent living facilities. The purpose of the study was to examine the relative and absolute reliability and the convergent validity of static standing balance performance using an accelerometer device**.**

**Methods:**

A total of 131 participants (85% female, mean age 80 ± 8 years) were included for the validity aim, and a subsample of 38 participants were enrolled in the reliability testing (89% female, mean age 76 ± 7 years). The root-mean-square (RMS) and normalized path length (NPL) for sway in antero-posterior (AP) and medio-lateral (ML) directions were calculated for different standing balance conditions. Test-retest reliability was assessed over two testing visits occurring 1 week apart using the intraclass correlation coefficient (ICC) for relative reliability, and the minimal detectable change (MDC) was calculated for the absolute reliability. Spearman’s rank correlation coefficient was used to test convergent validity at baseline between balance measurements and related mobility measures.

**Results:**

Reliability of balance performance using accelerometers was good to excellent with ICC values ranging from 0.41 to 0.83 for RMS sway and from 0.49 to 0.82 for NPL sway. However, the ICC during semi-tandem stance in A-P direction was 0.35, indicating poor reliability. The MDC of the sway measurements ranged from 2.4 to 9.4 for the RMS and 5.2 to 13.8 for the NPL. Balance measurements were correlated with mobility measurements.

**Conclusions:**

Using a portable accelerometer to quantify static standing postural control provides reliable measurements in community settings.

## Background

Normal aging is related to declines in different body systems such as cardiovascular, sensory, musculoskeletal, and cognitive function, all of which have been associated with increased risk of falling [1]. It is well documented that aging itself also is associated with a decline in muscle strength, balance, and functional mobility [2]. Maintaining postural stability is imperative for older adults to perform activities of daily living safely and independently within their society and thereby avoid falls [3]. Balance impairments are risk factors that contribute to mobility limitations and falls in older adults [1].

Because maintaining balance and mobility is important to successful aging, the assessment of balance is important for identifying older adults who are at high risk of falling, and also for developing appropriate exercise interventions to address any impairments. In order to achieve postural stability during standing, a person must be able to control the vertical projection of the center of mass within the base of support in the antero-posterior (AP, forward-backward) and medio-lateral (ML, side-to-side) directions. The measurement of body sway using an accelerometer around the waist can be used to record these movements of the center of mass, which is an advantage over wrist-mounted accelerometers commonly in use. Reliable and valid assessment instruments are necessary to obtain consistent and repeatable measurements for static standing balance. Currently, the most common methods to examine balance in clinical settings include observation-based measures; yet these measures have been shown to have examiner’s bias [4], suffer from floor and ceiling effects [5], cover limited aspects of balance, and often lack sensitivity to detect small changes in balance [6]. These drawbacks are major concerns for both clinicians and researchers who treat balance impairments and investigate the effectiveness of different balance interventions.

Over the last two decades, quantitative assessments of postural sway during standing using tools such as force plates have been used to assess postural stability and identify balance dysfunction in elderly population. Force plates have demonstrated good to excellent reliability for recording postural sway. However, because of the expense, space requirements, and lack of portability, their clinical utility in the community has been limited. Recent advances have provided an alternative quantitative method to assess balance that is inexpensive and portable by using body-worn accelerometers. Accelerometers are used to quantify postural sway during standing, and have been shown to have the ability to discriminate between test conditions that require different levels of postural control, between fallers and non-fallers, and young versus older adults [7–9]. Assessing balance by using accelerometers has been applied to different populations including people with Parkinson disease [10], stroke, children, and with community-dwelling older adults [11, 12]. Previous studies that have used accelerometers have demonstrated good to excellent test-retest reliability of postural sway measurements during the static standing balance [8, 12]. However, these accelerometer reliability studies were limited to clinical and lab settings, and had not been investigated outside in the community. Recently, a study by Saunders et al., found good to excellent test-retest reliability in using a tri-axial accelerometer to assess postural stability in people who live in independent living facilities [9].

To bridge the gap between expensive and immobile instruments and task-based measures, and by taking advantage of technological advancements in accelerometers, postural stability can be quantified portably and inexpensively outside of a lab setting. These tools can serve understudied populations, such as people living in community settings, who may have difficulty getting transportation to research labs. Therefore, the aim of this study was to establish the psychometric properties of balance measurements in older adults using an accelerometer.

## Methods

### Design and participants

This was an ancillary study to a cluster randomized clinical trial (RCT) that investigated the effect of two different group exercise programs conducted at their residence facility on walking ability, disability and self-reported function [13]. This study took place from April 2014 to May 2016. A subsample of 131 participants from the RCT were invited during their baseline assessment to take part in measurement validation. For test–retest reliability, a subsample of 38 participants returned 1 week later to take part in a retest session, also at their residence facility. This study has been approved by the Institutional Review Board of the University of Pittsburgh. Inclusion and exclusion criteria followed that of the parent study [13, 14].

### Balance Accelerometry

The accelerometer was developed as a part of the National Institutes of Health (NIH) Toolbox project as a balance measurement [15]. The dual axis accelerometer (ADXL213AE, with range of ±1.2 g and resolution of 1 mg; Analog Devices, Inc., Norwood, MA) is oriented to record acceleration of the body in both AP and ML axis. The acceleration is transmitted through Bluetooth transmitter to a laptop computer at 50 Hz. A custom written Labview program was used to acquire the data. The accelerometer was attached to the participant’s back at the level of the iliac crest using Velcro and gait belt.

### Study protocol

For assessment of test-retest reliability, participants repeated the following procedures on two separate occasions with 1 week apart. One week between testing sessions was chosen based on previous reliability studies [16–19], and to avoid the expected effect of an improvement in balance over the course of the intervention. Balance measurements included the following six standing conditions in order: (1) feet together on a firm surface with eyes open; (2) feet together on a firm surface with eyes closed; (3) feet together on a foam surface with eyes open; (4) feet together on a foam surface with eyes closed; (5) semi-tandem stance (1 foot halfway in front of the other) on a firm surface with eyes open; and (6) tandem stance on a firm surface with eyes open. All conditions were performed with the participant’s own pair of comfortable shoes. The foam surface that was used in the balance protocol is an AIREX® Balance Pad (Airex AG, Switzerland). For the semi-tandem and tandem stance conditions, the participants placed their feet according to their preference. Each condition was performed for a maximum of 30 s, and a rest of 30 s was provided between each trial.

### Outcome measures for the convergent validity

In order to examine convergent validity, the balance measurements at baseline were compared with mobility measures that were collected by in the parent study. These measures included the Six-Minute Walk Test (6MWT) [20], gait speed [21], Figure-of-8 Walk Test (F8WT) [22], Short Physical Performance Battery (SPPB) [23], and Gait Efficacy Scale (GES) [24].

### Six-minute walk test (6MWT)

The Six-Minute Walk Test (6MWT) is a well-validated measure of walking capacity. The test was included to measure walking endurance by calculating the maximum distance walked in 6 minutes, that includes rest time if needed [20]. Better performance is indicated by a greater distance covered during 6 minutes.

### Gait speed

Participants were asked to walk at their usual speed on an instrumented walkway [21]. Participants performed six passes and the average of the six passes was used in the analysis. Two practice trials were done before the real testing.

### Figure-of-8 walk test (F8WT)

The Figure-of-8 Walk Test (F8WT) measures motor skill in walking [22]. Participants walked a figure-of-8 pattern that was made by two cones with 1.5 m apart. Number of steps and time to finish the test were measured.

### Gait efficacy scale (GES)

The Gait Efficacy Scale (GES) is a 10 item scale used to address elderly’s perception of confidence during a challenging walking tasks such as walking over different surfaces, curbs, or stairs [24].

### Short physical performance battery (SPPB)

The SPPB was originally developed as a measure of physical performance for a longitudinal study of aging conducted by the National Institutes on Aging [23]. The SPPB measures three aspects of functional mobility: the time to perform five consecutive transfers from sitting to standing (chair stands), time to ambulate on level surfaces for 4 m, and the ability to stand with decreasing medial-lateral base of support. Scores from 0 to 4 are assigned to each of the tasks based on quartile scores of the timed chair stands and ambulation, and degree of difficulty of the standing balance test. A summary performance score is equal to the sum of the three sub-scores.

### Data analysis

#### Balance Accelerometry

The first and last 5 seconds of the recording were excluded from the data analysis in order to eliminate transient effects [25]. Using a custom written Matlab code, the acceleration data were lowpass filtered using a 4th order Butterworth filter with a cutoff frequency of 2 Hz. The Root Mean Square (RMS) and the Normalized Path Length (NPL) were calculated for both the antero-posterior (AP) and medio-lateral (ML) axis; a higher value indicates more sway. The RMS and NPL were computed as follows:

1 $$ RMS=\sqrt{{\frac{\left(\sum \limits_{j=1}^{N-1}{P}_j\right)}{N}}^2}\ \mathrm{mG} $$

2 $$ NPL=\frac{1}{t}\ {\sum}_{j=1}^{N-1}\ \left|{p}_{j+1}-{p}_j\right|\ \mathrm{mG}/\mathrm{s} $$

where *t* is the time duration, *N* is the number of time samples, and *p*_*j*_ is the acceleration data at time sample *j*. mG stands for milli-Gravitational acceleration, where 1 mG = 0.0098 m/s^2^.

### Statistical analysis

#### Overview

Data were analyzed using SAS software version 9.4 (SAS Institute, Inc., Cary, NC). Descriptive statistics of participant demographic characteristics were reported. The level of statistical significance was set at α ≤ 0.05 for all analyses.

#### Reliability

Test–retest reliability 1 week apart was estimated using intraclass correlation coefficients (ICC, model 3.1, two-way mixed-effects model) and 95% confidence intervals (95% CI). Absolute reliability of the balance accelerometry measurements was examined using the standard error of the measurement (SEM). The SEM is an estimate of the within-subject variability after repeated measures. The SEM was calculated using the sample standard deviation (SD) and the ICC as follows: SEM = SD √ (1 – ICC) [26]. In addition, the minimal detectable change (MDC) at the 95% level of confidence will be calculated for the outcome measures using the SEM values, as follows: MDC_95_ = SEM × 1.96 × √2 [26]. Bland–Altman plots were used to assess the agreement between test-retest measurements [27].

#### Validity

Face validity was examined by examining how body sway changed as the balance conditions became more difficult. These balance conditions were chosen to alter sensory feedback and reduce the base of support. A Friedman test was used to examine if there was a significant difference between the balance tests. Post hoc pairwise comparisons were performed with Wilcoxon signed ranks tests. The convergent validity was examined by calculating the correlation of balance measurements with the mobility measurements at the initial baseline assessment, using Spearman’s rank correlation coefficients.

## Results

Demographic and clinical characteristics of the study sample are summarized in Table 1. The subsample used for the reliability tests was 4 years younger than the total sample used for the validity examination. Compared with the total sample, the reliability subsample had a higher prevalence of diabetes, more comorbidities, and higher BMI.

**Table 1:** Demographic and clinical characteristics of participants

Variable	Validity Sample (***n*** = 131)	Reliability subsample (***n*** = 38)
**Age, years (SD)**	80.3 (7.7)	76.4 (6.5)
**Female, n (%)**	111 (85)	33 (87)
**Race-white n (%)**	110 (84)	31 (82)
**Married, n (%)**	28 (21)	6 (16)
**Education,** ^**a**^**n (%)**	70 (53)	18 (47)
**Chronic conditions**		
**Cardiac, n (%)**	24 (18)	9 (24)
**Musculoskeletal, n (%)**	115 (88)	33 (87)
**Visual/Hearing, n (%)**	104 (79)	24 (63)
**Diabetes, n (%)**	24 (18)	13 (34)
**Cancer, n (%)**	28 (21)	8 (21)
**Lung, n (%)**	41 (31)	13 (34)
**Total comorbidity**		
**> 3 conditions, n (%)**	40 (31)	16 (42)
**< 3 conditions, n (%)**	91 (69)	22 (58)
**BMI (kg/m**^**2)**^	28.3 (7.5)	31 (10.1)

^a^defined as attending at least some college

### Reliability

**Fig. 1:**
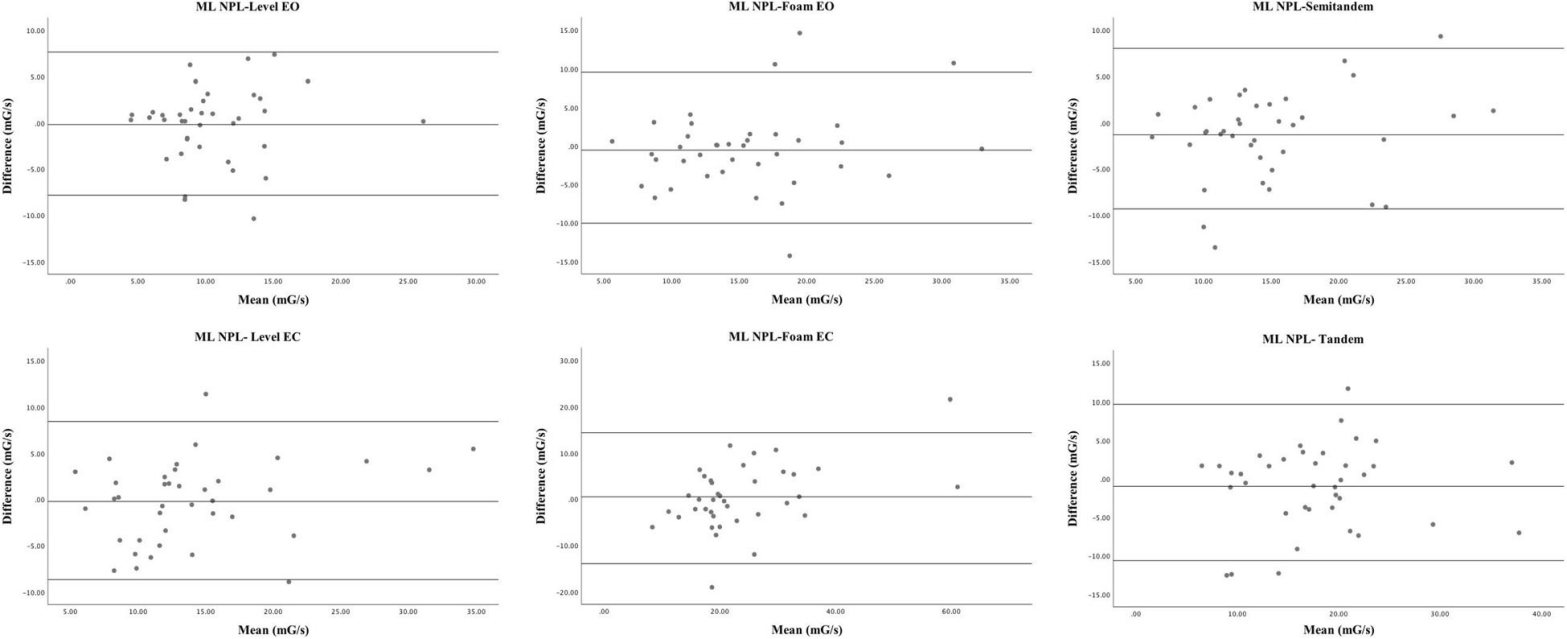
Bland–Altman plots representing mean differences and 95% limits of agreement between test and retest measurements for M-L NPL sway

A Wilcoxon signed-rank test showed no significant difference between the means of the test and retest sessions across all balance and strength measurements indicating no systematic bias was detected. The results of the test–retest relative reliability analyses, showing the ICCs values with their corresponding confidence intervals for balance accelerometry measurements (RMS and NPL sway in AP and ML axis) during all standing conditions are shown in Table 2. The ICCs were greater than 0.5 in most cases, except for the AP RMS and AP NPL measures when participants performed the semi-tandem stance condition, which demonstrated an increase in sway during the second test. After taking the average ICC across the different balance conditions, the ML NPL sway measures showed the highest ICC with an average of 0.73. The absolute reliability of all standing balance conditions, represented by the SEM and MDC, are reported in Table 3. Generally, eyes closed conditions have greater error than eyes open conditions for the same type of surface. Bland-Altman plots were similar in most of balance conditions, and a representative sample of the Bland-Altman plots for the ML NPL measure is shown in Fig. 1. The plots do not show any systematic change in difference between the test-retest values as the magnitude of the sway increases.

**Table 2:** Mean ± standard deviation (SD) of balance accelerometry measurements during the test and retest, *p*-values from the Wilcoxon signed ranks test, and reliability indicated by the intraclass correlation coefficient (ICC) and 95% confidence interval (*n* = 38)

Balance Conditions		Mean test ± SD	Mean retest ± SD	***p***-value	ICC (CI 95%)	ICC average
**AP RMS (mG)**	**Level EO**	7.51 ± 2.21	7.50 ± 2.40	0.95	0.81 (0.67–0.89)	0.61
	**Level EC**	8.57 ± 2.61	8.55 ± 2.27	0.92	0.58 (0.33–0.76)	
	**Foam EO**	7.70 ± 2.66	8.03 ± 2.70	0.18	0.77 (0.60–0.87)	
	**Foam EC**	11.70 ± 3.64	11.90 ± 4.30	0.89	0.63 (0.40–0.79)	
	**Semi-tandem**	7.91 ± 2.81	9.00 ± 3.51	0.09	0.41 (0.11–0.64)	
	**Feet tandem**	9.10 ± 4.73	8.65 ± 3.95	0.83	0.47 (0.18–0.68)	
**ML RMS (mG)**	**Level EO**	4.87 ± 2.02	4.43 ± 2.02	0.13	0.67 (0.47–0.82)	0.63
	**Level EC**	5.77 ± 2.30	5.94 ± 2.44	0.63	0.55 (0.28–0.74)	
	**Foam EO**	6.77 ± 2.93	7.11 ± 3.10	0.56	0.55 (0.28–0.73)	
	**Foam EC**	11.94 ± 5.42	12.07 ± 4.40	0.77	0.52 (0.25–0.72)	
	**Semi-tandem**	5.18 ± 2.03	5.49 ± 2.18	0.21	0.83 (0.71–0.91)	
	**Feet tandem**	5.54 ± 2.78	5.77 ± 2.57	0.46	0.71 (0.51–0.84)	
**AP NPL (mG/s)**	**Level EO**	10.28 ± 3.36	10.57 ± 3.18	0.17	0.66 (0.44–0.81)	0.64
	**Level EC**	14.53 ± 5.05	15.05 ± 5.88	0.70	0.66 (0.44–0.81)	
	**Foam EO**	11.24 ± 3.41	11.06 ± 3.57	0.80	0.71 (0.51–0.84)	
	**Foam EC**	17.62 ± 6.29	17.68 ± 6.14	0.93	0.82 (0.67–0.90)	
	**Semi-tandem**	13.44 ± 4.75	14.85 ± 5.50	0.23	0.35 (0.04–0.60)	
	**Feet tandem**	14.72 ± 6.41	15.63 ± 6.15	0.18	0.65 (0.41–0.80)	
**ML NPL (mG/s)**	**Level EO**	10.43 ± 4.66	10.61 ± 4.35	0.67	0.61 (0.37–0.78)	0.73
	**Level EC**	13.99 ± 7.34	14.22 ± 6.22	0.82	0.79 (0.64–0.88)	
	**Foam EO**	15.30 ± 7.07	15.87 ± 6.15	0.27	0.71 (0.50–0.83)	
	**Foam EC**	24.09 ± 13.00	23.61 ± 9.81	0.74	0.81 (0.67–0.90)	
	**Semi-tandem**	14.43 ± 6.84	15.74 ± 5.87	0.18	0.71 (0.51–0.84)	
	**Feet tandem**	17.31 ± 7.66	18.33 ± 7.27	0.46	0.73 (0.54–0.85)	

*EO* Eyes Open, *EC* Eyes Closed, *RMS* root-mean-square, *NPL* normalized path length, *AP* antero-posterior and *ML* mediolateral. RMS sway (mG), and NPL sway (mG/s)

**Table 3:** Absolute reliability indicated by standard error of measurement (SEM), and minimal detectable change (MDC)

Balance Conditions		SEM	MDC
**AP RMS (mG)**	**Level EO**	1.00	2.78
	**Level EC**	1.58	4.38
	**Foam EO**	1.28	3.55
	**Foam EC**	2.41	6.69
	**Semi-tandem**	2.42	6.73
	**Feet tandem**	3.19	8.76
**ML RMS (mG)**	**Level EO**	1.15	3.21
	**Level EC**	1.59	4.4
	**Foam EO**	2.02	5.61
	**Foam EC**	3.40	9.43
	**Semi-tandem**	0.86	2.44
	**Feet tandem**	1.43	3.99
**AP NPL (mG/s)**	**Level EO**	1.90	5.28
	**Level EC**	3.18	8.84
	**Foam EO**	1.87	5.20
	**Foam EC**	2.63	7.31
	**Semi-tandem**	4.13	11.45
	**Feet tandem**	3.71	10.29
**ML NPL (mG/s)**	**Level EO**	2.81	7.79
	**Level EC**	3.10	8.61
	**Foam EO**	3.55	9.86
	**Foam EC**	4.97	13.78
	**Semi-tandem**	3.36	9.31
	**Feet tandem**	3.87	10.75

*EO* Eyes Open, *EC* Eyes Closed, RMS root-mean-square, *NPL* normalized path length, *AP* antero-posterior and *ML* mediolateral. RMS sway (mG), and NPL sway (mG/s)

### Validity

**Fig. 2:**
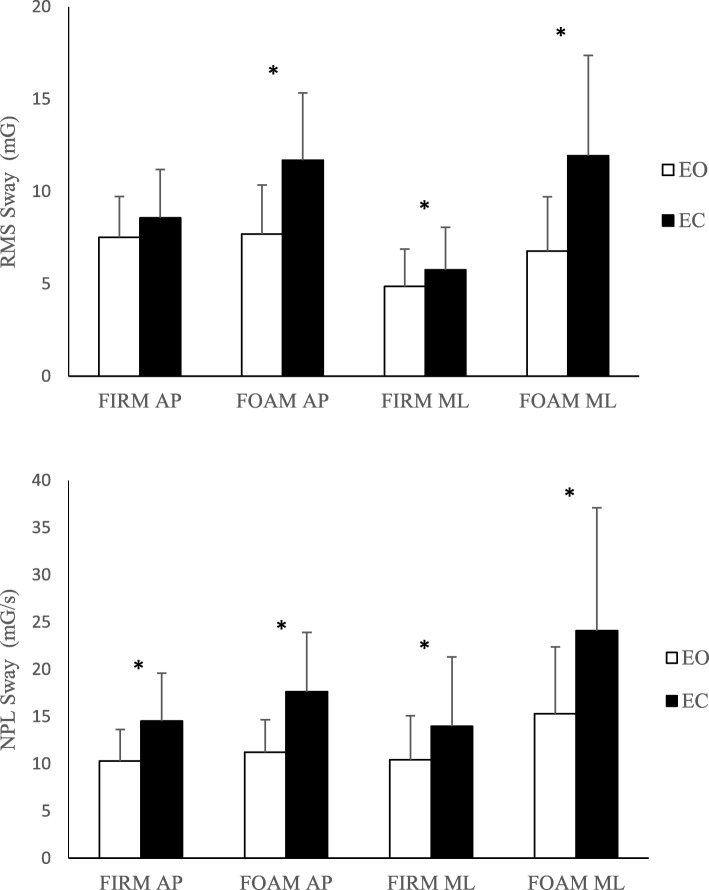
Effect of vision (Eyes Open: EO, and Eyes Closed, EC) and surface conditions (Firm, Foam) on root-mean-square (RMS, Top) and normalized path length (NPL, Bottom) sway acceleration for antero-posterior (AP) and mediolateral (ML) directions. (Error bars represent + 1 standard deviation); mG: milli-Gravitational acceleration, mG/s: milli-Gravitational acceleration divided by time duration; (*n* = 131). *: indicates significant difference with *p* < 0.001

In order to demonstrate face validity of the acceleration measures, we observed an increase in RMS and NPL sway in both directions as the difficulty of the balance conditions increased with eyes closed versus open, and foam versus firm surface (Fig. 2). The effect of vision (eyes open vs. eyes closed) was examined for each of the surface conditions. While standing on the firm surface, participants had a significant increase in sway for eyes closed compared with eyes open, in three out of the four acceleration measures (ML RMS, AP NPL, and ML NPL). On the foam surface, there was a significant increase in sway during eyes closed compared with eyes open for all four of the sway measures. Next, we tested the effect of surface (firm vs. foam) for each of the vision conditions. With eyes open, there was a significant increase in sway on foam compared with firm only for acceleration in the ML direction. However, with eyes closed, all four of the sway measures demonstrated an increase in sway during the foam condition.

Table 4 demonstrates the Spearman’s rank correlation coefficients between the RMS sway and NPL sway, and the SPPB, 6MWT, gait speed, F8WT, and GES. The table is ordered according to the mobility measurements that have the greatest number of significant correlations. The accelerometer sway measures had greater associations with the SPPB and GES functional measures compared with the timed tests of walking (F8WT, 6MWT, and gait speed). Additionally, better mobility test performance was associated with less sway. A sensitivity analysis was conducted to examine if adjusting for confounding variables such as BMI or age affected the associations. In most cases, there was minimal influence of BMI or age on the association between sway and the functional measures. However, BMI and age had the greatest influence on the associations between sway and the total SPPB score and 6MWT.

**Table 4:** Spearman rank correlation coefficients between balance accelerometry conditions and the Short Physical Performance Battery balance (SPPB_b) and total (SPPB_t) scores, Gait Efficacy Scale (GES), Figure of 8 Walk Test (F8WT), Six-Minute Walk Test (6MWT), and gait speed (*N* = 131)

Balance Conditions		SPPB_b	SPPB_t	GES	F8WT	6MWT	Gait speed
**AP RMS**	**Level EO**	−0.17*	− 0.24**	− 0.10	0.07	− 0.08	− 0.07
	**Level EC**	− 0.30**	− 0.24**	− 0.19*	0.09	− 0.06	− 0.10
	**Foam EO**	− 0.35**	− 0.33**	− 0.26**	0.21*	− 0.24**	− 0.22*
	**Foam EC**	− 0.24**	− 0.08	− 0.03	− 0.11	0.10	0.11
	**Semi-tandem**	−0.29**	− 0.26**	− 0.28**	0.02	− 0.09	−0.06
	**Feet tandem**	−0.33**	− 0.28**	− 0.25**	0.22*	− 0.08	− 0.05
**ML RMS**	**Level EO**	−0.40**	− 0.27**	− 0.15	0.12	− 0.12	− 0.24**
	**Level EC**	− 0.31**	− 0.15	− 0.15	0.02	− 0.01	− 0.10
	**Foam EO**	− 0.39**	− 0.32**	− 0.31**	0.23**	− 0.27**	− 0.28**
	**Foam EC**	− 0.15	− 0.04	− 0.09	−0.11	0.13	0.08
	**Semi-tandem**	−0.43**	− 0.30**	− 0.26**	0.20*	− 0.22*	−0.25**
	**Feet tandem**	−0.44**	− 0.42**	− 0.30**	0.24**	− 0.20*	− 0.27**
**AP NPL**	**Level EO**	−0.32**	− 0.30**	− 0.32**	0.27**	− 0.19*	− 0.24**
	**Level EC**	− 0.30**	− 0.25**	− 0.22*	0.15	− 0.10	− 0.12
	**Foam EO**	− 0.34**	− 0.35**	− 0.31**	0.21*	− 0.23**	− 0.20*
	**Foam EC**	− 0.19*	− 0.08	− 0.08	−0.08	0.12	0.10
	**Semi-tandem**	−0.28**	−0.32**	− 0.41**	0.31**	− 0.27**	− 0.19*
	**Feet tandem**	−0.34**	− 0.37**	− 0.30**	0.27**	− 0.14	− 0.17
**ML NPL**	**Level EO**	− 0.33**	− 0.11	− 0.17	0.08	− 0.02	− 0.12
	**Level EC**	−0.28**	− 0.06	− 0.12	0.01	0.05	−0.04
	**Foam EO**	− 0.35**	− 0.27**	− 0.27**	0.21*	− 0.20*	− 0.21*
	**Foam EC**	− 0.14	0.01	0.01	−0.16	0.20*	0.14
	**Semi-tandem**	−0.36**	−0.25**	− 0.18*	0.18*	− 0.03	−0.13
	**Feet tandem**	−0.25**	−0.29**	− 0.29**	0.27**	− 0.18*	−0.24**
**Number of significant correlations/total**		22/24	17/24	15/24	12/24	10/24	10/24

*indicates significant correlation coefficient *p* < 0.05

**indicates significant correlation coefficient *p* < 0.01

## Discussion

Across the six balance conditions, the sway measure that produced the greatest reliability was the normalized path length in the mediolateral direction, with ICC scores ranging from 0.61 to 0.81. In addition, some of the other sway measures had excellent reliability for specific test conditions. Only two measures had poor reliability: the AP RMS and AP NPL during semi-tandem stance. The current study had greater reliability coefficients compared with other published studies during the conditions on level surface with eyes open and closed [8, 28–30], possibly because the age range of our participants was larger, which may have produced greater intersubject variability.

Conversely, the current study had lower reliability than the study of Saunders et al. (2015) [9], who reported ICCs ranging from 0.77–0.93 for standing on a firm surface with eyes open and closed and ICCs from 0.76–0.95 for standing on foam surface. There are several possible reasons for the higher reliability in the Saunders study. In the Saunders study, they used the average of three trials for each balance condition, which would increase the ICC value compared to one trial in our study. It has been shown previously that test–retest reliability increased as the number of trials increase [31]. In the present study, to avoid fatigue of the elderly participants, only one trial was done. In addition, the retest session for the Saunders study was conducted within the same day. Evaluating test–retest reliability within-day has been shown to improve the ICC estimate as compared to between-day estimation [29]. Finally, they used a different foam surface than we used, and foam density and thickness can affect postural stability [32].

Our results for the NPL parameters were consistent with previous findings that used similar accelerometers for standing on a foam surface with eyes open and eyes closed in the AP direction [15, 33]. However, our results in these two conditions were slightly lower than results from Rine et al., (2013) [12], who reported an ICC of 0.88 for standing on foam with eyes open and 0.87 with eyes closed. In their study the retesting was done within the same day which could have yielded these higher ICC values.

The test–retest reliability during standing in semi-tandem and tandem stance was higher for the ML direction as opposed to the AP directions for both NPL and RMS sway. The semi-tandem and tandem stance conditions place more emphasis on the control of stance in the ML axis than AP, which seems to be more clinically relevant as ML sway has been associated with fall history [34]. Similarly, Moe-Nilssen et al. found higher ICCs for RMS acceleration in the ML (ICC = 0.84) than AP (ICC = 0.69) during standing on 1 foot where the base of support is more limited in the ML direction, thus providing support to our current findings [28].

The estimate of absolute reliability as indicated by the SEM and MDC provide researchers and clinicians with the ability to quantify the error during measurement and accurately estimate the true change on balance performance. Williams et al. 2016, reported similar MDC values for standing on a firm surface with eyes open and eyes closed using a triaxial accelerometer [29]. A smaller SEM and MDC indicates a more reliable measure. Larger SEM and MDC measures in this study may be attributed to: greater within-subject variability that is expected in older adults compared to other age groups; lack of a familiarization trial before test measurement, and not including more than one trial per session. In addition, the length of trial recording influences the reliability estimates with longer recordings associated with higher reliability. A duration of up to 120 s are suggested to reduce measurement error [35]. We used a 30-s sampling duration to match the abilities of older adults, who might not tolerate standing for an optimum duration.

Postural sway increased as the balance conditions became more challenging, thus demonstrating face validity of the accelerometer measurements. When somatosensory input was reduced by using a foam pad, the older adults generated greater body sway compared with standing on firm surface. Moreover, during conditions where visual inputs were absent, body sway increased as compared to eyes open conditions. Therefore, this has direct impact on older adults’ everyday lives, especially those with peripheral neuropathy or visual impairments who tend to have difficulty maintaining postural stability when walking on a carpeted floor or in a dark room. Our results are consistent with previous studies using a similar accelerometer [8, 25, 28]. In addition, the current results showed that the NPL sway in the AP axis when standing on foam with eyes closed was larger than the sway of healthy older adults with a mean age 47 years from a previous study that used a similar accelerometer, which further validates the measurements [33].

The Spearman correlation results showed a significant correlation in 17/24 of the balance parameters with the total SPPB score, and in 22 of the 24 of the correlations with the balance component of the SPPB, indicating convergent validity. To the best of our knowledge this is the first study that examined the correlation between balance accelerometry and the SPPB. Among all the included balance parameters, the highest correlation coefficients between sway measures and the balance component of the SPPB were the ML RMS sway during standing in semi-tandem and tandem stances (Spearman rho = 0.43 and 0.44, respectively). A simple explanation for this finding is that the semi-tandem and tandem balance conditions used for the accelerometer test mirrors the SPPB balance subtest. Previous studies showed similar results when comparing center of pressure measures using a force platform with clinical-based measures such as the SPPB [36, 37]. However, the moderate correlation indicates that different aspects of balance are being measured by the accelerometer-based measurements. The GES was significantly correlated with 15/24 of the sway measures. The highest value of correlation coefficients among the sway measures occurred in the foam, eyes open condition, and semi-tandem and tandem stances. These results indicate that individuals with greater sway had less confidence in their walking during everyday activities. Although, the correlation coefficients were significant, the strength of the relationship between the GES and sway measures was weak. This weak relationship could be explained by that the GES represents a person’s rating of their own confidence performing different walking-related tasks, whereas the balance accelerometry captures balance performance in standing only. A study that used another self-efficacy scale, such as the Activities-specific Balance Confidence (ABC) scale, which was highly correlated with the GES, showed a similar correlation between postural sway and the ABC scale [38].

The strengths of the current study are several. First, balance performance was quantified using a reliable method established in this specific population: i.e. older adults who live in independent living facilities. Second, we included various balance conditions that were designed to challenge and examine different balance sensory systems. Interpretation of the current findings should be considered in light of the following limitations. The sample in the current study was not randomly chosen from the parent study’s sample because this was an ancillary study to a multi-site cluster randomized trial, in which a subsample of the sites were chosen. However, baseline characteristics in our study were similar as compared to parent study. Another limitation is that we only included static standing balance conditions that examined one aspect of the balance system. Future research that includes dynamic balance tasks such as those in the Berg Balance scale could be done to explore the psychometric properties further. The reason for not including the dynamic conditions in this study is that older adults may not have tolerated a longer testing time, given that most of testing sessions were done after they finished testing from the parent study within the same day.

## Conclusions

The dual-axis accelerometer provides a feasible, reliable, and inexpensive method for testing standing balance in older adults. Among the included sway measures, the ML NPL measures demonstrated the highest test-retest reliability. Therefore, we recommend using these parameters to obtain a highly reliable measurement of sway in this population. Implementing the accelerometer technology may help investigators access understudied older populations living in independent living facilities, and will allow clinicians to examine objective measurements in real-life environments. Hopefully through the use of technology clinicians and therapists can prescribe interventions based on the individual’s objectively identified balance deficits.

## Data Availability

Data are available upon from the corresponding author on reasonable request.

## Abbreviations

6MWT: Six Minute Walk Test
AP: Anteroposterior
EC: Eyes Closed
EO: Eyes Open
F8WT: Figure of 8 test
GES: Gait Efficacy Scale
ICC: Intraclass Correlation Coefficient
mG: milli-Gravitational, (In relation to the Earth’s gravitational force)
mG/s: milli-Gravitational per second, (In relation to the Earth’s gravitational force)
ML: Mediolateral
NPL: Normalized Path Length
RMS: Root Mean Square
SEM: Standard Error of Measurement
SPPB: Short Physical Performance Battery
